# Homogenization of Mammalian Cultured Cells

**DOI:** 10.1100/tsw.2002.848

**Published:** 2002-06-13

**Authors:** John Graham

**Affiliations:** School of Biomolecular Sciences, Liverpool John Moores University, UK

**Keywords:** homogenization, cultured cells, homogenization media, nitrogen cavitation, cell cracker, ball-bearing homogenizer, Dounce homogenizer

## Abstract

Satisfactory homogenization of cultured cells is a necessary prerequisite to any fractionation schedule. Protocols are given for homogenization in iso-osmotic (A) and hypo-osmotic (B) media that should be broadly applicable to any cell type and to any subsequent fractionation procedure. Alternative procedures are also summarized in the Notes section, but detailed operation of some of the automated devices is beyond the scope of this short Protocol Article.

**METHOD TYPE:** extraction, isolation, purification and separation

**SUB METHOD TYPE:** centrifugation

## INTRODUCTION

Unlike an intact tissue such as rat liver, there are no definitive protocols for the homogenization of tissue culture cells that can be applied in all cases. The protocol depends crucially on whether the cells are grown as a monolayer or as a suspension culture. The former are much more easily disrupted than the latter. See Ref. [1] for a discussion of the methodology for homogenizing cultured cells.

The aim of the homogenization procedure must be to produce at least 90% cell breakage, reproducibly, under the mildest conditions. Clearly, homogenization under iso-osmotic conditions is an ideal which should be aimed for and an effective strategy for homogenizing monolayer cells under such conditions, based on the method of Marsh et al.[2], is given in Section A below.

For cells that fail to homogenize in iso-osmotic media unless severe or protracted homogenization procedures are used, it is probably more satisfactory to use hypo-osmotic swelling in order to render them more susceptible to lysis by the chosen homogenization technique. Iso-osmotic conditions are then re-established by adding a suitable osmotic balancer, once the cells have been disrupted. Generally most suspension culture cells require osmotic stress.

There are many such hypo-osmotic media such as 1 mM bicarbonate or any organic buffer at approx. 10 mM concentration. Divalent cations $Mg^{2+}$ or $Ca^{2+}$ at 1-2 mM may be added to protect the nuclei against lysis but this may also have an unwanted stabilizing effect on the plasma membrane. Sometimes sufficient osmotic stress to produce lysis can be achieved by using a reduced sucrose concentration of 0.1 M. One of the most successful strategies, devised by Goldberg and Kornfeld[3] uses a medium containing magnesium acetate and KCl and the protocol described in Section B below is adapted from Ref. [3].

In all cases the homogenization procedure must be carried out at 4°C. It should of course always be borne in mind that homogenization of any cell in any aqueous medium is bound to disturb the delicately balanced functions of the intact structure and to study any one subcellular compartment in the absence of others is an artificial situation. Nevertheless, as evinced by the plethora of scientific papers, the strategy of homogenization in an aqueous medium, followed by fractionation, is widely used to study subcellular processes. Even at low temperatures, due attention must be paid to detrimental effects of degradative enzymes that might be released from organelles during the homogenization and subsequent procedures. Some of these problems are addressed in Notes 1 and 2.

## HOMOGENIZATION OF MONOLAYER CELLS IN ISO-OSMOTIC SUCROSE

### Materials and Equipment

Phosphate-buffered saline (PBS)
Wash Medium: 0.25M sucrose, 10 mM triethanolamine-10 mM acetic acid, pH 7.8 (adjust to the correct pH with either triethanolamine or acetic acid, not HCl or NaOH)
Homogenization Medium (HM): Wash Medium containing 1 mM EDTA (see Notes 3–5)

Rubber policeman
Dounce homogenizer (tight-fitting, Wheaton Type A)

### Method

1. Use a near confluent monolayer.
2. Remove the medium and rinse the monolayer at least three times with PBS (at room temperature). Then wash the monolayer at least twice with Wash Medium (also at room temperature).
3. Add ice-cold HM to the dish (about 2 ml for a 9-cm dish) and scrape the cells into the medium with a rubber policeman. Do not try to produce a single cell suspension.
4. Transfer the crudely resuspended monolayer to a beaker on ice, washing the dish with a further 1 ml of HM to recover any remaining cells if necessary. Repeat the procedure for each dish.
5. If you end up with too large a volume, centrifuge the cells, and resuspend the pellet in a smaller volume of HM. Again do not try to produce a single cell suspension.
6. Homogenize the cells using 10–25 strokes of the pestle of a tight-fitting Dounce homogenizer. Observe the suspension after 10 strokes under the phase contrast microscope. Continue homogenization until about 90% of cells have been broken (see Notes 6–9 for alternative methods of homogenization).

## HOMOGENIZATION OF CELLS IN A HYPO-OSMOTIC MEDIUM

### Materials and Equipment

Swelling medium (SM): 15 mM KCl, 1.5 mM magnesium acetate (MgOAc), 1 mM dithiothreitol (DTT), 10 mM Hepes-KOH, pH 7.5 (see Note 10)
Osmotic balancer (OB): 375mM KCl, 22.5 mM MgOAc, 1mM DTT, 220 mM Hepes-KOH, pH 7.5
Hepes-buffered saline (HBS)
Dounce homogenizer (tight-fitting, Wheaton Type A)

### Method

1. Wash the cells twice in HBS to remove all traces of the culture medium.
2. Suspend the cells in 10 ml of SM and allow them to swell on ice for 10 min.
3. Centrifuge the cells and remove sufficient supernatant to leave a volume equivalent to 3.5x that of the cell pellet.
4. Homogenize in a tight-fitting Dounce homogenizer and then add 1/5th of the volume of OM (see Notes 6–9 for alternative methods of homogenization).

## NOTES

1. It is common to guard against possible protein hydrolysis in the homogenate by including a cocktail of protease inhibitors in HM: for example, 1 mM phenylmethylsulphonyl fluoride (PMSF) and 2 μg/ml each of antipain, leupeptin, and aprotinin. These however are not effective against the activity of glycosidases or lipoxygenases. The latter are instrumental in the production of potentially damaging lipid hydroperoxides. See Ref. [4].
2. In certain cases it may be possible to use a nonaqueous homogenization medium that will prevent the removal of surface proteins from organelles and the solubilization of certain enzymes. Ethylene glycol and formamide have been used successfully for the isolation of nuclei[5] and minimize loss of certain RNA polymerases but the isolation of other organelles is less well established and moreover, is compromised by the subsequent requirement to assay enzymes. More often than not, nonaqueous media pose more problems than they solve.
3. The buffer is critical for the success of this method, no substitute is totally satisfactory.
4. One of the major problems with cultured cells is the severity of the shearing forces required to effect efficient cell disruption. The greater the number of strokes of the pestle, the greater the possibility of causing nuclear rupture. Release of DNA, even from a few nuclei will cause severe aggregation of material: this will lead to the loss of large amounts of material into the nuclear pellet. It may therefore be advisable to add DNAase I to the homogenate to minimize this problem.
5. Proteins from the cytoskeleton may also form a gel-like structure and cause aggregation of subcellular components, this can be alleviated by the inclusion of 10–20 mM KCl, if this is compatible with effective homogenization.
6. Generally Potter-Elvehjem homogenizers are less efficient than the Dounce type for cultured cells.
7. Repeated aspiration and ejection of a cell suspension through the narrow orifice of a syringe needle may provide a useful alternative.
8. There are several commercially available devices, which can make the shearing process more reproducible. **In the Cell Cracker** the cell suspension is repeatedly passed (using two syringes) through the narrow annulus between a ball and a metal block. This is now regarded as one of the most reliable and gentle methods of homogenizing cultured cells (see Ref. [6]). **In the Stansted Cell Disruptor** the cell suspension is forced, under high pressure from a piston or compressed nitrogen through a narrow orifice. The big advantage of this device is that the shear force is applied once to the entire cell suspension rather than repeatedly as in manually operated versions.

9. **Nitrogen cavitation** involves the exposure of a stirred cell suspension to nitrogen gas at about 800 psi (5,516 kPa) at 4°C for about 15 min within a stainless-steel pressure vessel. The suspension is then forced through a needle valve by the gas pressure, at which point cell rupture occurs by a combination of the sudden expansion of gas dissolved within the cytosol and the formation of bubbles of nitrogen gas in the medium. The method is successful with all types of cell. Gas equilibration parameters (time and pressure) and solution composition need to be tested to optimize the results.
10. The ionic composition of this medium tends to avoid any "gel" formation by cytoskeletal proteins and by homogenizing in a small volume, the organelles, which are released, are protected from hypo-osmotic shock by the cytosolic proteins.

## ACKNOWLEDGEMENTS

The author and TheScientificWorld wish to thank Axis-Shield PoC, AS, Oslo, Norway for their kind permission to adapt OptiPrep™ Application Sheet S6 in the preparation of this Protocol Article.
